# Optimising Care for Haemophagocytic Lymphohistiocytosis in District General Hospitals: Best Practice Insights and Review of Literature

**DOI:** 10.7759/cureus.80957

**Published:** 2025-03-21

**Authors:** Aadithiyavikram Venkatesan, Rita Deb, Rahim Nadeem Ahmed, Steven Vidgeon, Sundar Ashok, Stella Kotsiopoulou

**Affiliations:** 1 Department of Critical Care Medicine, Croydon University Hospital, London, GBR; 2 Department of Haematology, Croydon University Hospital, London, GBR

**Keywords:** critical care centre, haemophagocytic lymphohistiocytosis (hlh), infection associated hlh, organ failure from sepsis, primary lymphoma

## Abstract

Haemophagocytic lymphohistiocytosis (HLH) is a hyper-inflammatory syndrome characterised by widespread, uncontrolled T cell and histiocyte activation with accompanying cytokine storm. It can be inherited or acquired, with acquired forms triggered by infection, malignancy or autoimmune disease. There is high mortality, with patients commonly requiring critical care support. HLH is a relatively rare condition (though it is likely underdiagnosed), and there is an incomplete understanding of its pathogenesis, diagnosis and optimal management. As such, HLH presents a challenge to the critical care physician, particularly in a non-specialist centre. In our District General Hospital (DGH) intensive therapy unit, we experienced an unusually high volume of HLH cases in a short period. By presenting this case series and our improvement strategies, we aim to distil key lessons to other centres to improve the understanding of HLH for critical care physicians.

There were seven confirmed cases of HLH between January 2022 and August 2024. We analysed each case, in turn, documenting the initial symptomatology, blood test results, including ferritin, and when specialist teams such as haematology and critical care became involved. We then noted when HLH treatment was started and the outcome of each case. We found that implementing a protocol to encourage early requests of an HLH blood test panel and, thereafter, early review and input by the local haematology teams, as well as critical care input for any patient with suspected HLH, improved patient outcomes: two patients died during admission pre-intervention. However, none of the patients died within their admission after our interventions. We also found that pharmacy input to ensure the emergency stock of medications used to treat HLH allowed more prompt administration of HLH treatment.

Although the cornerstone of management for the critical care physician includes supportive care and organ support, HLH-specific treatments targeting the underpinning inflammation and the cytokine storm are also pivotal to controlling the disease process. Early escalation to the haematology team based on abnormally high ferritin levels and early critical care input was the cornerstone of the pathway we developed in our hospital. This system that has been developed will stand to be useful in other DGHs where HLH remains an unfamiliar condition that carries a high mortality rate.

## Introduction

Haemophagocytic lymphohistiocytosis (HLH) is a hyperinflammatory syndrome characterised by widespread and uncontrolled T cell and histiocyte activation with accompanying cytokine storm. HLH can be driven by inherited mutations, often affecting CD8+ T cells or natural killer cell cytolytic pathway genes [1,2]. These inherited forms are estimated to account for 25% of HLH presentations and are generally in paediatric populations [2]. In adults, acquired/secondary HLH pre-dominates instead. These syndromes are associated with a trigger, commonly infection (particularly viral), malignancy (haematological being the most common) or autoimmune disease [1,3]. However, ongoing research does suggest that these individuals may also harbour predisposing mutations overlapping with primary HLH genes [3,4].

Clinical features are that of high fever, cytopenia, organomegaly and derangement of certain biomarkers, particularly ferritin and triglycerides [5]. As the disease progresses, multi-organ failure ensues, and there is high mortality, estimated at 40% [6]. Prevalence is difficult to discern as HLH is likely underdiagnosed due to unfamiliarity and diagnostic overlap with other hyperinflammatory syndromes [7,8]. Recent retrospective studies done in the United States showed an incidence of more than 16,000 cases of diagnosed HLH over 13 years in young adults and children [9]. Diagnosis relies on clinical judgement and the use of scoring systems based on clinical, biochemical and histological factors. At present, the HLH 2004 diagnostic criteria and HScore are the most utilised diagnostic tools [8-10]. HLH has many overlapping signs and symptoms with sepsis, including pyrexia and hyperferritinaemia. The diagnosis of HLH relies on the combination of various other parameters, as outlined in the HScore criteria. Due to its high mortality rate and rapid development of multi-organ failure, critical care physicians are likely to encounter HLH patients. At present, there is a lack of consensus on HLH classification, diagnosis and management. As such, HLH presents a distinct challenge, particularly in the non-specialist critical care unit.

Here, we describe our experiences with seven of these cases and present a brief review of the literature, by which we hope to share our learning and recommendations for the care of HLH patients and provoke further discussion of this complex, under-recognised and life-threatening disease. This study also aims to highlight the important parameters to look for patients with suspected HLH to aid in early diagnosis, management and escalation. This allowed us to develop a diagnosis and management protocol, which can be easily adapted in other hospitals to aid and promote awareness of the condition.

## Materials and methods

These diagnosed cases were all adults (age >18 years old) presented to our critical unit between June 2022 and August 2024. No exclusion criteria were considered. Electronic data were accessed for all these patients. No obvious missing data were identified during data collection.

Case 1

A 29-year-old female patient presented to the hospital with bilateral neck swelling. Lymphadenopathy was identified on an ultrasound scan and subsequently biopsied for histological analysis. This confirmed a diagnosis of stage 4B anaplastic large cell lymphoma. Over the first three days of her admission, she developed abdominal pain and fever, with tachycardia and tachypnoea. A pleural effusion was identified on her chest radiograph. Sepsis was diagnosed, and appropriate therapy was initiated in the form of intravenous antibiotics and fluids. At this point, HLH was suspected due to a ferritin level of 2,096 ug/L, lactate dehydrogenase (LDH) level of 218 and a triglyceride level of 3.8 mmol/L. On day 6 of admission, her HScore was 145 (correlating with a 16%-24% probability of HLH).

Given the high suspicion of HLH developing, methylprednisolone was started, and a critical care review was sought. As she did not currently require organ support, she was not admitted to the critical care unit. However, her HLH biomarkers continued to worsen. On day 10, her HScore was 200; hence, anakinra was commenced, and methylprednisolone was switched to dexamethasone as per the advice of the haematology team. On day 11, she developed hypokalaemia (3.1 mmol/L) and was admitted to the high-dependency unit (HDU). Due to her increasing HLH score, the anakinra dose was up-titrated, and treatment of the diagnosed T cell lymphoma was started with a cyclophosphamide, doxorubicin, vincristine, etoposide, and prednisolone chemotherapy regime. On day 14, the patient acutely deteriorated, requiring vasopressors to maintain blood pressure and entering respiratory distress, requiring intubation. Despite best attempts, the patient continued to deteriorate, entering multi-organ failure with refractory hypotension. On day 15, the patient suffered a cardiac arrest. In the context of treatment-refractory multi-organ failure, cardiopulmonary resuscitation was not attempted.

Case 2

A 22-year-old male patient presented to the hospital with a three-day history of fever and bilateral knee joint pain. He had a nine-day admission one week before this with fever, headaches, back pain, and unexplained weight loss with diffuse lymphadenopathy, where he was diagnosed with an acute hepatitis A infection (IgM positive). During this previous admission, he had haematology input for raised HLH markers (ferritin as high as 19,987 ug/L) but did not meet the criteria for HLH. Preliminary differential diagnoses on his re-admission included a non-specific viral illness or a rheumatological disorder. Initial blood tests revealed a stable transaminitis compared to his results from the previous admission (alkaline phosphatase 243 U/L, alanine aminotransferase 693 U/L and elevated ferritin level 9,479 ug/L). Over the first three days of his admission, he became hypotensive and tachycardic, with temperatures reaching 39.5°C. A further working diagnosis of infective endocarditis was considered after an echocardiogram raised suspicion of mitral valve vegetation, so empiric antibiotics were administered. Computed tomography (CT) scan revealed mild hepatosplenomegaly with axillary lymphadenopathy. Knee joint aspiration was also carried out to elucidate the cause of his arthralgia/joint swelling, which produced a scanty sample with minimal cell count.

On day 2, his ferritin level increased to 14,860 ug/L, with an incomplete HScore of 141 (correlating with a 16%-25% probability of HLH). On day 3, anakinra was started after a bone marrow biopsy taken on that day, which revealed macrophages with haemophagocytic activity, aligning with a diagnosis of HLH. It was felt HLH may have been triggered by either infective endocarditis, septic arthritis or potential adult-onset Still’s disease. On day 8, he was admitted to the intensive therapy unit (ITU) due to worsening hypotension and ongoing pyrexia. Methylprednisolone was added to his HLH treatment regime of anakinra. His HScore at this moment was 221 (i.e., a 96%-98% probability of HLH). By the end of day 8, etoposide was also started as per the haematology team as an additional HLH treatment. On day 10, the patient was transferred to a tertiary referral centre specialising in HLH for further management.

Case 3

A 79-year-old male patient with a past medical history of ischaemic heart disease, hypertension, rheumatoid arthritis, prostate cancer and hypothyroidism was admitted due to worsening bilateral leg ulcers, which had been present for six weeks. Initially starting as a rash, this had progressed in the community to deep ulceration with exposed bone. On admission to the hospital, he was treated for infected ulcers with antibiotics. He was found to be pancytopenic, and this was presumed to be secondary to infection or methotrexate usage (which was discontinued). The aetiology of his ulcers remained unclear.

On day 10 of admission, the patient deteriorated with a new cough and development of pyrexia, along with chest radiograph findings suggestive of hospital-acquired pneumonia. Following advice from the microbiology team, broad-spectrum antibiotics were started. The patient underwent a skin biopsy of his leg ulcers on day 20. This was non-diagnostic, although suggestive of either a vasculitic process or pyoderma gangrenosum. Considering this, prednisolone 30 mg was commenced on day 24 to treat rheumatoid vasculitis. Due to persistent pyrexia, raised inflammatory markers and a worsening clinical picture despite antibiotics, HLH was first considered on day 24 of admission. The calculated HScore was only 102, and therefore, treatment for sepsis continued, though daily HLH biomarker screening was undertaken. The patient deteriorated further on day 29 with new-onset atrial fibrillation with rapid ventricular response and worsening acute kidney injury. A marked increase in ferritin was noted, now at 27,000 ug/L. Along with accompanying increases in other HLH biomarkers, this gave an HScore of 175, so a presumptive diagnosis of HLH was made. As such, methylprednisolone (1 g IV) was commenced. The patient was reviewed by the critical care outreach team in light of his HLH diagnosis, but at that time, they concluded the patient did not require HDU admission. Further screens were sent to investigate the trigger of HLH, including a bone marrow aspirate and trephine (BMAT), which revealed haemophagocytosis.

Anakinra was introduced on day 31 due to rising aspartate aminotransferase and triglycerides, and steroid therapy was switched to dexamethasone as the patient had developed confusion about methylprednisolone. Despite improving HLH biomarkers, the patient underwent a critical deterioration on day 35 with circulatory shock and type 1 respiratory failure. He was transferred to HDU for the commencement of organ support.

The patient arrived in HDU in respiratory distress, with profound hypotension, reduced consciousness and metabolic acidosis. He was started on vasopressor therapy, along with amiodarone. A transfusion of red cells was given.  It was decided the patient would not be for intubation and ventilation or cardiopulmonary resuscitation due to established rheumatoid pulmonary fibrosis and advanced frailty. Despite aggressive resuscitation, the patient continued to deteriorate, and hence, the next day, it was decided to withdraw active treatment. The patient passed away on day 36 of admission, less than 24 hours after escalation to HDU. Post-mortem, blood cultures were taken on admission to HDU, and *Escherichia coli* and Proteus grew.  Results of the BMAT obtained on day 30 became available post-mortem, which revealed a likely mantle cell lymphoma with 20% marrow involvement.

Case 4

A 36-year-old male patient with a background of epilepsy was admitted to the hospital with fever and malaise, having recently been diagnosed with stage 4 T cell/histiocyte-rich B cell lymphoma-like transformation of nodular lymphocyte predominant Hodgkin lymphoma. He was initially treated for neutropenic sepsis. On day 2 of this admission, due to pancytopenia, pyrexia and splenomegaly, following a haematology review, it was decided to screen for HLH markers. HScore was 198 with ferritin levels of 21,000 ug/L. The patient was diagnosed with HLH secondary to lymphoma. He was given methylprednisolone and was also commenced on his previously planned cyclophosphamide, doxorubicin, prednisone, rituximab and vincristine chemotherapy regime for his newly diagnosed lymphoma. Advice was sought from the local HLH specialist centre, after which methylprednisolone was switched to dexamethasone based on the 1994 HLH Protocol.

The patient was admitted to HDU locally for monitoring purposes following changes to local policy advising admission to HDU for all HLH patients on first-line therapy. While on HDU, the patient required no organ support. Etoposide was commenced on day 7 of admission due to rising HLH markers despite first-line HLH treatment with steroids and anakinra. The patient responded well, with improving inflammatory markers, HLH markers and resolution of fever. He stepped down from HDU on day 9. Epstein-Barr virus (EBV) polymerase chain reaction samples sent on day 8 of admission showed a high viral load. This may have also contributed to the development of HLH in this patient. It was determined that concurrent R-CHOP and etoposide therapy would treat his Epstein-Barr viraemia, and indeed, his viral count decreased to 208 international units/mL at discharge. The patient continued to improve and was discharged after 37 days. He continued lymphoma treatment on an outpatient basis.

Case 5

A 19-year-old female patient initially presented to the hospital with right temporal headache, fever, coryzal symptoms and an incidental finding of iron deficiency anaemia (haemoglobin 55 g/L, ferritin 4 ug/L). An initial diagnosis of sinusitis was made, for which she received antibiotics, as well as an intravenous iron infusion, as per the advice of the haematology team, following a negative thalassaemia and haemolytic screen. Twelve days later, she presented with ongoing high fevers up to 40.6°C and was discharged with a further course of antibiotics. However, two days after this, she reattended with recurrent fever, myalgia, dry cough and cervical lymphadenopathy. On this third presentation, she was hypotensive, with a ferritin of 8,610 ug/L and a worsening neutropenia. The patient was reviewed by the haematology team, and daily HLH testing and Hscore monitoring were initiated since HLH was now suspected.

On day 2 of admission, she was commenced on a three-day course of intravenous methylprednisolone and daily subcutaneous anakinra, given her HScore was now 182 (probability of 70%-80%). She underwent a bone marrow biopsy on day 4, which revealed red cell aplasia and haemophagocytosis in keeping with HLH, with no clonal B or T cells and no excessive blasts. The rheumatology team reviewed her and subsequently advised that a rheumatological cause of her HLH was unlikely. They agreed with the haematology team, which suspected that the trigger was either an infection or a reaction to the intravenous iron transfusion she had received just over two weeks ago. She continued to undergo daily HLH biomarker monitoring, alongside a magnetic resonance imaging scan of her head and neck to assess her lymphadenopathy, as well as a positron-emission tomography CT scan, both of which were unremarkable. On day 11, given the sustained improvement in her HLH biomarkers and symptomatic improvement, she was discharged on daily anakinra and a weaning course of oral steroids. She continued to be monitored locally as an outpatient.

Case 6

A 34-year-old female patient had recently returned from a trip to Nigeria. Two weeks later, she was admitted to hospital with a four-day history of swinging fevers, malaise, polyarthralgia, bilateral frontal headache, lower abdominal pain, diarrhoea and exertional dyspnoea. She had not taken any malaria prophylaxis. On admission, she was hypotensive and had a blood film confirming *Plasmodium falciparum* infection with a parasitaemia of 21.5%. She was started on intravenous artesunate and antibiotics and admitted to ITU as per the protocol for the management of severe malaria. The haematology team were consulted on admission due to her severe thrombocytopenia with a platelet count of 15. That evening, she became unresponsive and required emergency intubation. A CT scan of the head was normal. She was acidotic, with deranged liver and renal function and ongoing thrombocytopenia. Antibiotics were escalated as per the advice of the infectious diseases team at our local tertiary centre, and she was commenced on haemofiltration.

On day 2, her ferritin result was 43,809 ug/L. The haematology team advised calculating her HScore, which was 230 (which correlates with a 96%-98% probability of HLH). It was believed she had developed HLH secondary to falciparum malaria. They advised to start anakinra but to hold off on steroids pending discussion with the Infectious Diseases team, given the unknown risk of steroid treatment in patients with severe malaria and sepsis. On day 3, advice from the local tertiary centre specialising in HLH was sought; they advised to commence two days of intravenous immunoglobulin (IVIG) and a three-day course of methylprednisolone. On day 3, she was also prepared for extubation; however, she had a low Glasgow coma score (E1VTM3 E1VTM3, meaning eye response 1, verbal response not testable and motor response 3) despite 90 minutes off sedation. The thoughts from the nearby infectious diseases team were that this was concordant with severe cerebral malaria and, hence, recovery would be slow. They advised with risks and benefits in mind, concluding that continuing steroids for her HLH would be appropriate even in the context of cerebral malaria. After seven days in our unit, she was transferred to the ITU at the nearest tertiary centre, specialising in HLH for ongoing care.

Case 7

A 61-year-old female patient presented with a four-day history of intermittent epigastric pain radiating to her back and night sweats. She had a background of secondary myelofibrosis transformed from essential thrombocythaemia, for which she was taking ruxolitinib twice daily. On admission, she had a raised C-reactive protein (CRP) level and was anaemic with a haemoglobin of 59 g/L. Other cell counts were borderline low, including her neutrophils. Her blood film was reviewed by the haematology team, who advised her to treat it as neutropenic sepsis as there was a risk of functional neutropenia. The film was in keeping with her known diagnosis of myelofibrosis, but due to the presence of some blast cells, transformation to acute myeloid leukaemia or progression of the disease could not be ruled out without immunophenotyping.

A CT scan of her abdomen and pelvis was arranged, which revealed calculous cholecystitis. Both the gastroenterology and surgical teams conducted a review during her admission and recommended the continuation of antibiotic treatment, along with a referral for cholecystectomy following her discharge. Blood cultures during this time were positive for *Staphylococcus hominis,* which the microbiology team deemed a likely contaminant. However, the patient had started spiking temperatures, so her antibiotic regime was escalated and then again on day 7 when her fevers did not cease. Repeat blood tests revealed a raised LDH level and a dropping neutrophil and platelet count. On day 8, her peripheral blood immunophenotyping results showed pancytopenia with occasional circulating blasts from two abnormal myeloid progenitor populations.

On day 16 of her admission, due to persistent pyrexia and high CRP, she underwent a repeat CT scan of her abdomen and pelvis, now also including her thorax. This showed bibasal consolidation of her lungs with pleural effusions and unchanged appearances of her gallbladder or pancreas. An "infiltrative bone marrow process" was appreciated in the form of numerous lytic lesions in her ribs. A myeloma screen was sent, and the haematology team advised, given her progressing cytopenia and ongoing fevers, to begin monitoring for HLH with an HLH biomarker screen and to arrange a BMAT. Her HScore on day 19 was 228 (ferritin now 6,006 ug/L compared to 1,869 ug/L on admission), correlating with a 96%-98% probability of HLH.

On day 20, she was admitted to ITU as she had newly developed type 1 respiratory failure and was persistently tachycardic. A repeat chest radiograph showed pulmonary oedema and worsening pleural effusions. Her preliminary bone marrow results showed she had >40% blasts, confirming the transformation of her myelofibrosis to an acute leukaemia. The case was discussed with the haematology team at the tertiary centre, which provided care for her myelofibrosis. They advised to start anakinra to treat HLH, likely secondary to the acute leukaemia transformation. She was subsequently transferred to them to start chemotherapy promptly.

Table 1 presents a summary of all cases.

**Table 1:** Clinical summary of all cases HLH: haemophagocytic lymphohistiocytosis; ITU: intensive therapy unit; CHOEP: cyclophosphamide, doxorubicin, vincristine, etoposide, prednisolone; UCLH: University College London Hospitals; IVIG: intravenous immunoglobulin; B/L: bilateral

Cases	Age/gender	Presenting complaints	Working diagnosis	HLH provisional diagnosis	Treatment started	What treatment was given	Peak ferritin (ug/L)	Peak triglycerides (mmol/L)	Cell lines affected	ITU admission	Level of ITU support	Prognosis
Case 1	29/female	Lymphadenopathy	Lymphoma	Day 5	Day 6	Dexamethasone + anakinra + CHOEP regimen	2,800	7.5	RBC, WBC	Day 35	3	Deceased
Case 2	22/male	Fever, arthralgia	Stills disease	Day 3	Day 4	Methylprednisolone + anakinra + etoposide	38,000	2.6	RBC, WBC	Day 7	2	Transferred to UCLH
Case 3	79/male	B/L leg ulcers	Rheumatoid vasculitis	Day 24	Day 29	Methylprednisolone + anakinra + dexamethasone	27,000	4.3	RBC, WBC	Day 35	3	Deceased
Case 4	36/male	Fever, lymphoma	Neutropenic sepsis	Day 2	Day 2	Methylprednisolone + anakinra + dexamethasone	28,000	3.7	RBC, WBC	Day 4	1	Discharged
Case 5	19/male	Fever, coryzal symptoms	Sinusitis	Day 2	Day 3	Methylprednisolone + anakinra	8,610	1.77	RBC, WBC, platelets	Day 2	2	Discharged
Case 6	34/female	Fever, polyarthralgia	Malaria	Day 3	Day 3	Methylprednisolone + IVIG + anakinra	43,809	6.3	RBC, WBC, platelets	Day 1	3	Transferred to UCLH
Case 7	61/female	Epigastric pain	Neutropenic sepsis	Day 19	Day 10	Methylprednisolone + anakinra	6,006	5.3	RBC, WBC	Day 10	1	Discharged

## Results

Diagnosis of HLH is incredibly challenging, as highlighted in the literature and within our seven cases. The use of established criteria such as HScore can be helpful, as can the identification of hyperferritenaemia. In particular, ferritin levels of >4,000 ug/L increase the likelihood of HLH, and levels >10,000 ug/L should prompt higher suspicion of HLH, potentially warranting immediate treatment. Sepsis resistant to appropriate therapy should prompt further screening for possible HLH. In case 3, acute deterioration was presumed secondary to HLH. Post-mortem, it became apparent that the patient had *E. coli* and Proteus bacteraemia. This highlights that while the focus on sepsis may delay an HLH diagnosis, it can also complicate the course of HLH, particularly given the reliance on immunosuppressive treatments to treat HLH. There must be high vigilance for sepsis in HLH as a confounder, trigger and complication. We believe early admission to HDU for close monitoring and ease of immediate intervention for HLH patients is appropriate, even before the need for organ support. In cases 1 and 3, both patients underwent rapid unforeseen and ultimately terminal deteriorations in the ward setting.

Following this, local policy was changed to mandate admission to HDU of all patients diagnosed with HLH on first-line therapy, as seen in case 4. In case 3 particularly, there was a prolonged period before HLH was considered and treated. This was partly due to diagnostic uncertainty regarding the initial presentation, in contrast to patients 1 and 4, who had early diagnoses of lymphoma, a common HLH trigger. Suspicion of HLH should still be maintained even before an obvious trigger can be found, and treatment should not be delayed. Case 3 (rheumatoid vasculitis/sepsis/mantle cell lymphoma) and case 4 (EBV and Hodgkin’s lymphoma) highlight that triggers may be difficult to identify and may be multi-factorial. Cases 2 and 4 highlight that despite the high mortality of HLH, with early detection and intervention, good outcomes are possible. Case 4, in particular, highlights what we believe should be the aim of HLH care: early intervention before organ support is necessary, with the involvement of specialist teams and intensive monitoring/supportive care. As outlined in the above cases, patient outcomes from HLH presentations in our hospital varied from death during admission (cases 1 and 3) to discharge home without ever requiring organ support (case 4).

**Figure 1:**
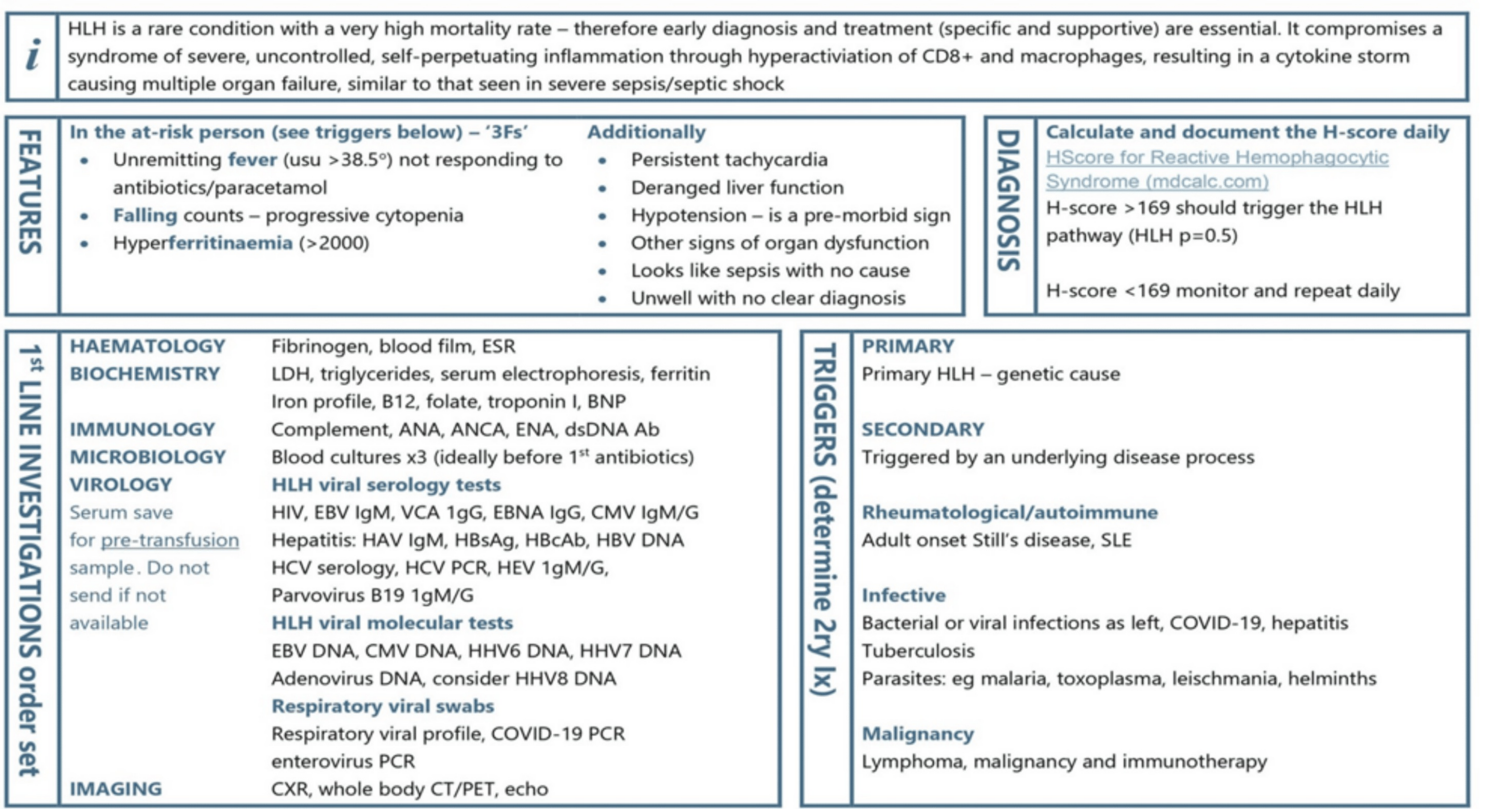
Diagnostic pathway for HLH Image credits: This is an original image created by the authors Steven Vidgeon, Sundar Ashok, Rahim Nadeem Ahmed, and Aadithiyavikram Venkatesan HLH: haemophagocytic lymphohistiocytosis

**Figure 2:**
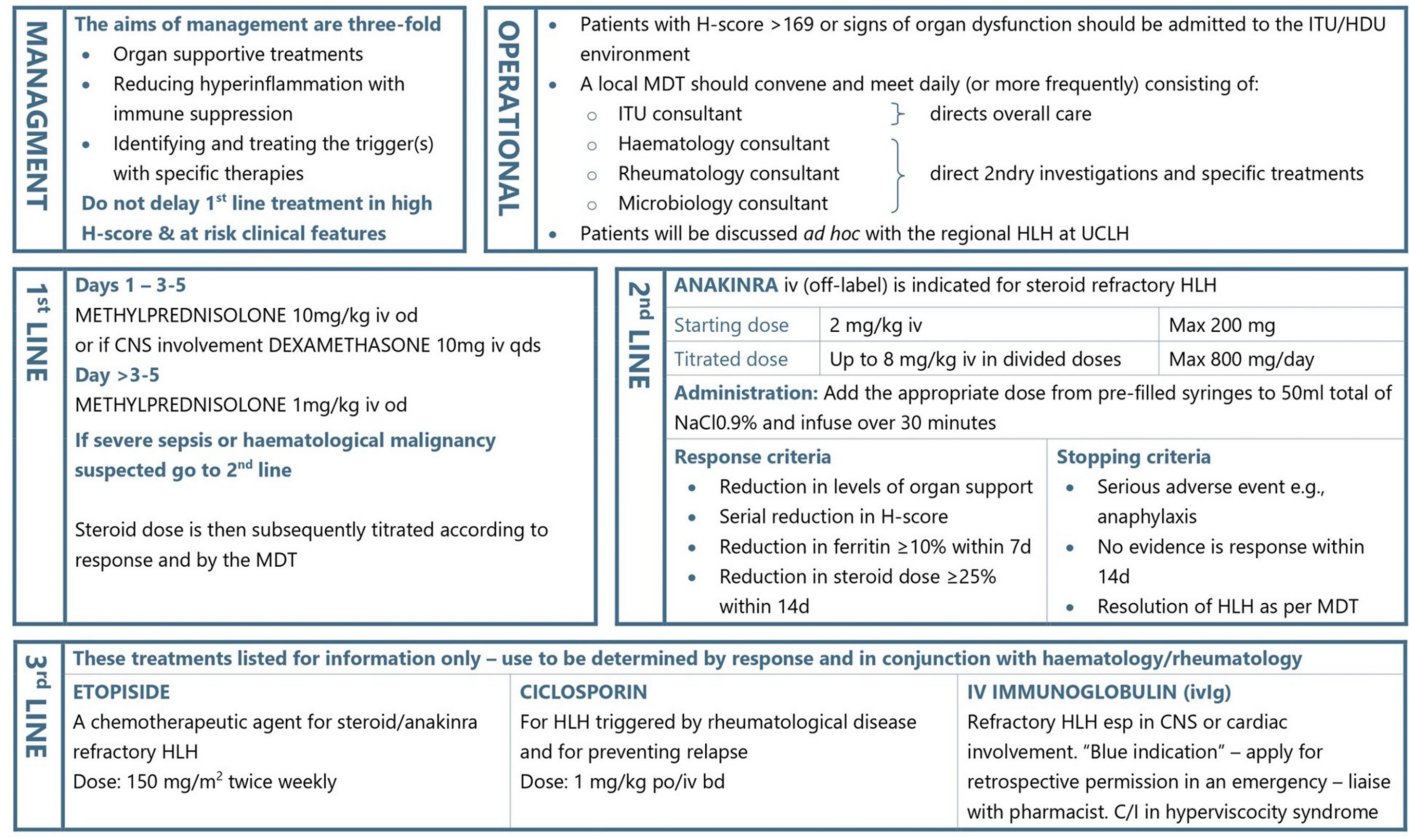
Therapeutic pathway for HLH Image credits: This is an original image created by the authors Steven Vidgeon, Sundar Ashok, Rahim Nadeem Ahmed, and Aadithiyavikram Venkatesan HLH: haemophagocytic lymphohistiocytosis

These cases were discussed at the ITU Clinical Governance meeting, following which it was agreed to implement a more robust screening and action process to tackle this condition in an ITU setting. We developed a management protocol in line with National HLH treatment guidelines (University College London Hospitals) and local haematology/rheumatology team input, which was aimed at educating the clinical staff in our ITU. We also started measuring serum ferritin levels upon ITU admission, and any alarmingly high levels (>5,000 ug/L) prompted immediate liaison with the haematology team. Moreover, early discussions with the haematology and rheumatology teams were advised in any situation where HLH was considered a potential diagnosis. Our newly developed trust HLH pathway is previewed in Figures 1, 2.

We also worked with our ITU pharmacy team to help arrange for HLH medications, such as anakinra, to be available on the weekend for emergency use, preventing delays to HLH treatment once the condition was diagnosed.

The above improvements had a positive effect on the early diagnosis and treatment of HLH in our ITU, as evidenced by cases 4-7, whereby patients were promptly diagnosed due to early screening, timely involvement of specialist teams and rapid administration of HLH treatment.

Due to the change in clinical practice, there is now more awareness and prompt escalation when HLH is suspected. This is very prudent from a District General Hospital (DGH) standpoint due to the lack of resources such as specialist clinical teams and advanced respiratory supports, such as extracorporeal membrane oxygenation (ECMO), for rapidly deteriorating patients.

This system that has been developed will stand to be useful in other DGHs where HLH is still an unheard-of condition that carries a high mortality.

## Discussion

HLH in the critical care setting presents multiple challenges in both the identification and management of patients. HLH patients are commonly labelled with another diagnosis [2], or they may present to critical care with an established HLH diagnosis or develop HLH during their critical care stay. Diagnosis is complex, with many of the cardinal features of HLH also present in other hyperinflammatory conditions such as sepsis. HLH is likely underdiagnosed in the critical care population, with estimates of up to 77% of cases going undiagnosed by retrospective analysis [8,10,11]. Early contact with relevant speciality teams (usually haematology and/or rheumatology) can aid in appropriately investigating high-suspicion cases [12]. A detailed clinical history, including inherited inflammatory disorders and malignancies, is helpful in the diagnosis of HLH and in elucidating the likely trigger.

A screening tool often suggested to improve the diagnosis of HLH in critical care is the identification of hyperferritinemia [2,6,10,11], with varying suggested cutoffs ranging from 500 to 5,000 ug/L as a threshold prompting further investigation of HLH [6,13,14]. However, there is continuing controversy regarding this practice, with some, such as Schram et al., questioning the specificity of hyperferritinemia as a screening tool for HLH [15]. Other disorders with high ferritin levels can be discerned from HLH using a combination of clinical and biochemical characteristics outlined in the Histiocytic Society diagnostic recommendations for HLH [8]. The HScore is based on a single-centre retrospective study of rheumatological patients, which has external validation. While selection bias in the study is highlighted, it provides a high probability of patient diagnosis with HLH when utilised. It calculates the probability of HLH based on nine clinical and laboratory parameters. However, it has not been validated for use in critical care patients [6]. Currently, there is ongoing research into novel biomarkers for detecting HLH, such as soluble interleukin-2 receptors [15]. Overall, while diagnostic approaches are developing, ultimately, the diagnosis of HLH requires careful consideration of clinical context and integration of various clinical and biochemical factors. Increased awareness and vigilance of the possibility of HLH may facilitate earlier diagnosis and treatment.

For the critical care physician, the cornerstone of care for HLH patients is supportive care and organ support [16-19]. This does not differ greatly from that of other cytokine storm/multi-organ failure syndromes. Particular attention should be paid to monitoring and/or prophylaxis for developing infections, given the reliance on immunosuppressive medications [2,18,20]. Also, there is a common need for extensive blood product replacement [21].

The most frequent reasons for ITU admission in HLH patients in a large retrospective analysis were acute respiratory failure (35%), shock (29%) and multi-organ failure (10%) [22]. There are no published data regarding the choice of vasopressor for the management of circulatory shock associated with HLH. Ventilatory strategies should be guided by the identified respiratory pathology, with acute respiratory distress syndrome being common [21]. There is one reported case using ECMO successfully for an adult with HLH [23]. Many centres have taken the approach of applying therapies successfully in other cytokine storm-driven pathologies. Indeed, this has shown some success in the case of anakinra [24] and IVIG [25]. Case reports have described the successful use of cytokine adsorption therapies along with first-line anti-HLH medications where a cytokine removal column was added into the circuit of haemodiafiltration, resulting in an immediate decrease in inflammatory parameters. However, there is currently insufficient evidence for this as an HLH-specific therapy [26,27].

From our literature review, we summarise the consensus recommendations in Table 2.

**Table 2:** Consensus list of recommendations from literature review HLH: haemophagocytic lymphohistiocytosis; SIRS: systemic inflammatory response syndrome; EBV: Epstein-Barr virus

Recommendations from the literature review
HLH should be considered when there is SIRS/hyperinflammatory syndrome with an unclear source or where there is a lack of response to appropriate treatment[16]
HLH should be considered when SIRS or multi-organ failure is combined with other features of HLH, e.g., organomegaly and cytopenia
There should be high suspicion for HLH in at-risk groups (e.g., particularly those with haematological malignancy, red flag infections, such as EBV, etc.) [8]
Screening for hyperferritinaemia may be useful to help raise suspicion of HLH and prompt further investigation [6,13], but there is no universally accepted cut-off [5]
The HLH 2004 criteria or HScore can be used to aid identification of patients, though this has not been validated in critical care populations[17]

Despite the valuable insights provided by this study, several limitations should be acknowledged. First, the sample size was relatively small (seven cases), which may limit the generalisability of the findings to a broader population. Additionally, another limitation is the retrospective design, which prevents the establishment of cause-and-effect relationships between various parameters. High ferritin levels are commonly associated with sepsis and liver failure, and at times, it may provide a false positive impression to diagnose HLH. It is prudent to consider and evaluate further parameters of HLH in the patient to arrive at a higher probable diagnosis by utilising the HScore. Lastly, long-term follow-up was not analysed as part of this study, as the priority was to ascertain early diagnosis and treatment. Further research is required to establish national guidelines for hospitals lacking advanced organ support facilities and long-term follow-up of patients' post-HLH treatment.

## Conclusions

The implementation of a pathway/guideline that prompted the early multidisciplinary assessment (i.e., haematology and/or rheumatology, critical care outreach and ITU/HDU) of patients who were suspected to be developing HLH had a positive impact on the outcome for patients presenting thereafter. Recognition of a raised ferritin was pivotal to this. We encourage other DGHs to consider the development of such HLH guidelines and the local education of clinical staff as to this rare but potentially life-threatening diagnosis.
